# Trends in Ophthalmic Surgery Among Older Patients in Japan (Fiscal Years 2018 to 2022): A National Claims Database Study on Surgical Shifts and the COVID-19 Pandemic's Impact

**DOI:** 10.7759/cureus.85808

**Published:** 2025-06-11

**Authors:** Yoshiaki Kabata

**Affiliations:** 1 Ophthalmology, Jikei University School of Medicine, Daisan Hospital, Tokyo, JPN

**Keywords:** covid-19, glaucoma, ndb open data japan, older adults, ophthalmic surgery, surgical trends

## Abstract

Background: Japan has the highest proportion of older adults globally, making its healthcare data crucial for understanding worldwide aging trends. This study investigates trends in ophthalmic surgeries among older Japanese patients using the National Database of Health Insurance Claims (NDB), with a particular focus on the impact of the COVID-19 pandemic.

Methods: This retrospective database analysis utilized publicly available aggregated data from the Japanese NDB Open Data portal, managed by the Ministry of Health, Labour and Welfare (MHLW), covering fiscal years (FY) 2018 to 2022. We examined annual trends in the number of cataract surgeries (procedure code K282), glaucoma surgeries (K268), and vitrectomies (sum of K272-K281), stratified by three age groups based on the Japanese Society of Gerontology classification: 65-74 years ("pre-old"), 75-89 years ("old"), and ≥90 years ("super-old"). Glaucoma surgery (K268) encompassed iridectomy, outflow reconstruction, trabeculectomy, tube shunt implantation without a plate, tube shunt implantation with a plate, and trabecular micro-bypass stent implantation combined with phacoemulsification. Laser procedures were excluded. We performed a descriptive analysis of surgical counts and calculated the percentage change between FY2018 and FY2022. Population data for the same age groups were obtained for comparison.

Results: Cataract surgery demonstrated an increasing trend across all age groups from FY2018, with a transient decrease in FY2020 attributable to COVID-19-related postponements, followed by renewed growth. Vitrectomy also exhibited a temporary decline in FY2020. Glaucoma surgeries displayed a consistent upward trend, although the increase in FY2020 was marginal. While the population aged 65-74 decreased to 80% of its 2018 level, the populations aged 75-89 and ≥90 years grew to 101% and 123%, respectively. In contrast, increases in surgical volumes far outpaced population growth. Glaucoma surgery volumes, for instance, increased to 169%, 168%, and 170% of 2018 levels across the respective age groups. Vitrectomy increased to 103%, 113%, and 139%, and cataract surgery to 111%, 113%, and 115%. Among glaucoma procedures, outflow reconstruction, tube shunt implantation with a plate, and especially trabecular micro-bypass stenting with phacoemulsification showed marked increases.

Conclusion: Ophthalmic surgeries generally increased among older adults in Japan from FY2018 to FY2022, with a transient decrease in FY2020 linked to the COVID-19 pandemic. The significant rise in specific glaucoma procedures, particularly minimally invasive glaucoma surgery (MIGS) combined with cataract surgery, signals an evolution in surgical practices. These trends have important implications for healthcare resource planning and surgeon training.

## Introduction

In 2021, Japan recorded the world's highest proportion of individuals aged 65 and older, constituting 28.7% of its total population [1], a demographic trend projected to continue [2]. As the global leader in population aging, Japan's healthcare landscape offers valuable insights for other nations facing similar demographic shifts. Analyzing Japanese medical data can inform policy development and the redesign of social security systems worldwide [2].

The Ministry of Health, Labour and Welfare (MHLW) established the National Database of Health Insurance Claims and Specific Health Checkups of Japan (NDB) under the Act on Assurance of Medical Care for Older Patients. Accumulating data since 2008, the NDB is one of the most comprehensive national healthcare databases globally [3]. Since Japan's universal healthcare system covers most medical care, the NDB, which includes data from inpatient (including Diagnosis Procedure Combination (DPC)), outpatient, pharmacy, and dental claims, accurately reflects national medical trends and informs government healthcare policy [3]. The NDB Open Data Japan portal provides aggregated summary data for public use.

Ophthalmic surgery, often performed under local anesthesia, is feasible even for the very elderly. Cataract surgery, in particular, is commonly performed on nonagenarians and centenarians [4-6]. A prior study using NDB Open Data examined ophthalmic surgery trends across all age groups from fiscal year (FY) 2014 to FY2020 [7]. The COVID-19 pandemic, with the first case identified in Japan in January 2020, prompted a nationwide postponement of non-emergency surgeries following the state of emergency declared in April 2020. This disruption likely impacted ophthalmic surgery volumes, particularly in FY2020. Because older adults faced higher mortality risks from COVID-19 [8], they may have been more inclined to postpone medical visits and elective surgeries. Therefore, examining surgical trends specifically around the pandemic period (FY2018-FY2022) and focusing specifically on the older population is crucial.

This study aimed to analyze five-year trends (FY2018-FY2022) in the volume of major ophthalmic surgeries-specifically cataract surgery, glaucoma procedures, and vitrectomy-performed on older adults in Japan. Using aggregated data from the NDB, the study also examines how these trends were influenced by the COVID-19 pandemic and age-related demographic shifts.

## Materials and methods

This study was a retrospective, descriptive analysis based on publicly available, aggregated data from the NDB Open Data Japan portal [3]. We analyzed data covering FY2018 to FY2022 (April 1, 2018, to March 31, 2023). The analysis included aggregated counts of specific ophthalmic procedures performed on patients within three age groups, following the classification by the Japanese Society of Gerontology: 65-74 years ("pre-old"), 75-89 years ("old"), and ≥90 years ("super-old").

We identified the annual number of specific ophthalmic surgeries using the following Japanese medical procedure codes: cataract surgery, code K282 (phacoemulsification and intraocular lens implantation); vitrectomy, sum of codes K272 through K281; glaucoma surgery, code K268 (this comprehensive code includes iridectomy, outflow reconstruction (trabeculotomy), trabeculectomy, tube shunt implantation without a plate (e.g., EX-PRESS®), tube shunt implantation with a plate (e.g., Ahmed®, Baerveldt® implants), and trabecular micro-bypass stent implantation combined with phacoemulsification (e.g., iStent®)). Consistent with the approach of Tanito [9], laser treatments for glaucoma were not included. This single code represents the entire combined procedure, thus preventing the same surgical event from being counted twice under both the glaucoma code (K268) and the cataract surgery code (K282).

The primary outcomes were the annual number of cataract surgeries, vitrectomies, and total glaucoma surgeries (K268) performed within each age group from FY2018 to FY2022. Secondary outcomes included trends for each specific glaucoma procedure under K268. Data on the annual number of procedures for each specified code were extracted from the NDB Open Data spreadsheets for each fiscal year and age group. A descriptive analysis was conducted to assess trends over the five-year period. All aggregated data used in this study were downloaded from the official NDB Open Data portal provided by the Japanese Ministry of Health, Labour and Welfare.

To quantify changes, we calculated the percentage change in the number of surgeries between FY2018 and FY2022 using the formula Percentage Change (%) = (Number in FY2022 / Number in FY2018) × 100. For context, we obtained Japanese national population estimates for the same age groups for 2018 and 2022 from the Statistics Bureau of Japan [1] and calculated the percentage population change using the same formula. Given the descriptive nature of the study and the use of aggregated data, no advanced statistical modeling was performed.

This study utilized publicly accessible, anonymized, aggregated data and was therefore deemed exempt from formal ethical review and the need for informed consent by the Institutional Review Board of Jikei University School of Medicine. The study adhered to the principles of the Declaration of Helsinki.

## Results

**Figure 1:**
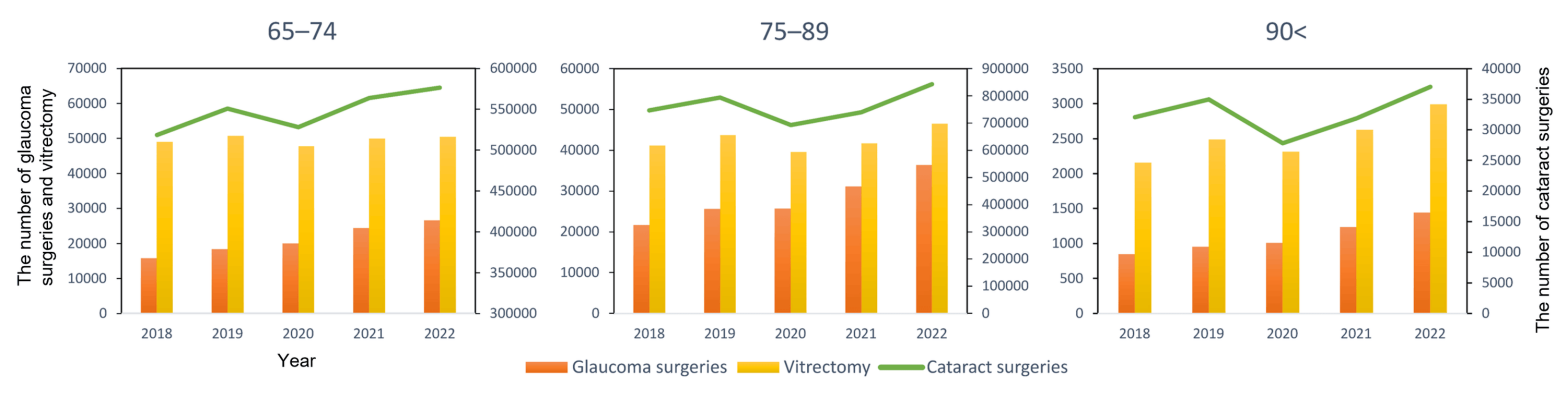
Trends in ophthalmic surgeries in older adults (FY2018-FY2022) Annual number of vitrectomies and glaucoma surgeries (left y-axis, bars) and cataract surgeries (right y-axis, lines) performed in Japan for age groups 65–74, 75–89, and 90 years and over, based on NDB Open Data. FY = Fiscal Year (April 1 to March 31). The Japanese government declared a state of emergency for COVID-19 in April 2020 (start of FY2020).

Figure 1 illustrates the annual number of vitrectomies, glaucoma surgeries, and cataract surgeries from FY2018 to FY2022, stratified by age. Cataract surgery numbers generally increased from FY2018 but showed a transient decrease in FY2020 before resuming an upward trend. Vitrectomy also demonstrated a temporary decrease in FY2020; however, an overall increasing trend was observed in the 75-89 and ≥90 age groups, while the 65-74 group remained relatively stable, excluding the FY2020 dip. Glaucoma surgeries exhibited a consistent increasing trend across all age groups, although growth was marginal between FY2019 and FY2020.

**Figure 2:**
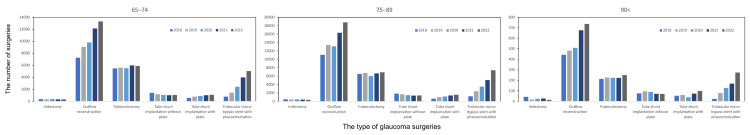
Trends in specific glaucoma surgeries (FY2018-FY2022) Annual number of specific glaucoma surgery types (iridectomy, outflow reconstruction, trabeculectomy, tube shunt implantation without plate, tube shunt implantation with plate, and trabecular micro-bypass stent with phacoemulsification) performed in Japan for age groups 65–74, 75–89, and 90 years and over. FY = Fiscal Year (April 1 to March 31).

Figure 2 illustrates the trends for specific types of glaucoma surgeries. Notably, procedures involving outflow reconstruction, tube shunt implantation with a plate, and trabecular micro-bypass stent implantation combined with phacoemulsification demonstrated substantial increases across most age groups. Conversely, iridectomy and tube shunt implantation without a plate showed decreasing trends.

Table 1 shows the population aged 65-74 years decreased to 80% of its 2018 level by 2022, while the 75-89 group slightly increased (101%), and the ≥90 group grew significantly (123%). In contrast, surgical volumes generally outpaced population changes. Glaucoma surgeries increased markedly across all groups: 169% (65-74), 168% (75-89), and 170% (≥90) relative to FY2018. Vitrectomy increases were 103% (65-74), 113% (75-89), and 139% (≥90). Cataract surgery increases were 111% (65-74), 113% (75-89), and 115% (≥90).

**Table 1:** Japan's population in 2018 and 2022 and the number of ophthalmic surgeries in fiscal years 2018 and 2022 stratified by three age groups Glaucoma surgeries are the sum of the above six surgeries. The increase was particularly significant in trabecular micro-bypass stent with phacoemulcation in all age groups.

	Aged 65–74			Aged 75–89			Aged 90<		
	2018	2022	change (%)	2018	2022	change (%)	2018	2022	change (%)
Population of Japan	9368000	7535000	80%	6932000	7030000	101%	2000000	2450000	123%
Iridectomy	339	311	92%	457	342	75%	42	13	31%
Outflow reconstruction	7262	13294	183%	11081	18784	170%	442	736	167%
Trabeculectomy	5465	5868	107%	6502	6901	106%	213	249	117%
Tube shunt implantation without plate	1393	1035	74%	1842	1403	76%	75	70	93%
Tube shunt implantation with plate	541	1081	200%	617	1552	252%	53	98	185%
Trabecular micro-bypass stent with phacoemulsification	768	5019	654%	1184	7415	626%	23	274	1191%
Glaucoma surgeries	15768	26608	169%	21683	36397	168%	848	1440	170%
Vitrectomy	49035	50492	103%	41080	46479	113%	2156	2990	139%
Cataract surgeries	518476	576324	111%	746849	842877	113%	32087	37020	115%

Examining specific glaucoma procedures, the increases from FY2018 to FY2022 were substantial. Outflow reconstruction increases were 183% (65-74), 170% (75-89), and 167% (≥90). Tube shunt implantation with plate increases were 200% (65-74), 252% (75-89), and 185% (≥90). Trabecular micro-bypass stent with phacoemulsification increases were 654% (65-74), 626% (75-89), and 1191% (≥90).

## Discussion

This study revealed generally increasing trends in cataract, vitrectomy, and glaucoma surgeries among Japanese patients aged 65 and older between FY2018 and FY2022. However, a noticeable decrease or stagnation in surgical volume occurred in FY2020, coinciding with the peak of the first COVID-19 wave and the declaration of a national state of emergency in April 2020. This suggests that the pandemic led to the postponement of many ophthalmic procedures.

Our findings align with Wada et al. [7], who reported decreased numbers of cataract and vitreoretinal surgeries in FY2020, while glaucoma surgeries showed less impact. They hypothesized that cataract and some vitreoretinal surgeries were considered more elective than glaucoma surgery, where delays risk irreversible vision loss [7]. Our results, extending to FY2022, confirm the rebound in cataract and vitrectomy numbers post-FY2020. Interestingly, while the overall glaucoma surgery trend was upward, the stagnated growth between FY2019 and FY2020 suggests that even these procedures were affected, possibly due to older patients' heightened concerns about COVID-19 exposure [8] or temporary resource reallocation within hospitals.

A key finding of this study is the shifting landscape within glaucoma surgery. Procedures involving outflow reconstruction (including ab interno trabeculotomy), tube shunt implantation with a plate (for complex glaucoma), and particularly trabecular micro-bypass stenting combined with phacoemulsification - a form of minimally invasive glaucoma surgery (MIGS) - showed significant growth [10-12]. Conversely, traditional iridectomy and tube shunt implantation without a plate (EX-PRESS®) declined. The EX-PRESS® device, once popular, faced concerns regarding complications and efficacy comparable to trabeculectomy, potentially leading surgeons toward other options like trabeculectomy, valved/non-valved implants, or MIGS, depending on the target intraocular pressure (IOP) [11-14].

The dramatic ascent in combined phacoemulsification and trabecular micro-bypass stent procedures reflects the global trend towards MIGS, especially for patients with mild-to-moderate glaucoma [11,12,15,16]. In Japan, ab interno trabeculotomy (classified under outflow reconstruction) has also gained popularity for its cost-effectiveness and favorable IOP reduction [13,15]. The increasing prevalence of combined cataract and glaucoma surgery [17] synergizes with the high volume of cataract procedures in the aging population and is a major driver of the observed rise in glaucoma surgeries. While formal cost-effectiveness studies for MIGS in the very elderly in Japan are limited [17,18], the potential benefit of reducing medication burden is particularly valuable in this population.

This study has several limitations inherent to claims-based research. The NDB Open Data provides aggregated counts, lacking patient-level clinical details such as disease severity, visual acuity, or comorbidities. Furthermore, claims coding may be subject to institutional or individual variations, potentially introducing inaccuracies. The dataset does not capture the complex multidisciplinary management often required for older surgical patients. Therefore, our findings reflect broad procedural trends rather than detailed clinical practice patterns or outcomes. Furthermore, as our study is a descriptive analysis of aggregated national counts, we did not perform inferential statistical tests to assess the significance of trends. Such tests would be inappropriate without access to patient-level data to account for variance and confounding variables. Therefore, the significance of the observed trends is interpreted based on the magnitude of change and graphical analysis rather than formal statistical proof.

## Conclusions

This analysis of the Japanese National Database highlights an overall increase in cataract, glaucoma, and vitreous surgeries among older adults from FY2018 to FY2022, interrupted by a transient decline in FY2020 associated with the COVID-19 pandemic. Within glaucoma surgery, there was a marked shift toward procedures involving outflow reconstruction and tube shunts with plates, alongside a dramatic rise in combined phacoemulsification and trabecular micro-bypass stenting, signaling a paradigm shift toward MIGS. These trends reflect advancements in surgical technology, evolving treatment paradigms, and the growing healthcare demands of Japan's super-aged society. Continued monitoring of these trends is essential for healthcare planning, resource allocation, and optimizing ophthalmic surgical training to meet the needs of an aging global population and enhance their quality of life.
